# The complete and circular genome of nitrogen-fixing *Mesorhizobium* sp. AaZ17, isolated from the root nodule of *Astragalus armatus* growing in a lead- and zinc-abandoned mine in Morocco

**DOI:** 10.1128/mra.01234-24

**Published:** 2025-02-27

**Authors:** Soufiane Alami, Zohra Chaddad, Mouad Lamrabet, Kaoutar Kaddouri, Hanaa Abdelmoumen, Mustapha Missbah El Idrissi

**Affiliations:** 1 Center of Plant and Microbial Biotechnology, Biodiversity and Environment, Faculty of Sciences, Mohammed V University in Rabat 226283 , Rabat, Morocco; The University of Arizona, Tucson, Arizona, USA

**Keywords:** *Mesorhizobium*, *Astragalus armatus*, genome sequencing, GridION, phylogenetic analysis

## Abstract

In this study, we sequenced the genome of *Mesorhizobium* sp. strain AaZ16, a nitrogen-fixing rhizobial species isolated from the root nodules of *Astragalus armatus* growing wild in a lead- and zinc-rich mine tailings in the High Atlas, Morocco. This study reveals the genomic characteristics of the root microsymbiont.

## ANNOUNCEMENT

Here, we report the complete genome of *Mesorhizobium* sp. strain AaZ16, nodulating *Astragalus armatus* in the heavy metal-rich Zaida mine tailings (32.8349022, –4.9570041), Morocco (1). *Astragalus armatus* root nodules obtained by trapping method from the mine tailings were surface disinfected using 75% (v/v) absolute ethanol for 2 minutes and then crashed in 500 µL sterile distilled water. A loop was streaked on yeast-mannitol agar (YMA), in Petri plates, and incubated at 28°C for a week. Pure single bacterial colonies obtained were cultured four times on YMA and stored at +4°C. Strain AaZ16 produces siderophores and ACC deaminase (2).

Total genomic DNA was extracted from the liquid culture in TY medium using the PureLink genomic DNA minikit (Invitrogen by Life Technologies) and quantified using a NanoDrop ND2000/2000 c. The sequencing was conducted on the Oxford Nanopore GridION device, and the library was prepared using the Rapid Sequencing gDNA Barcoding Kit (SQK-RBK004) and sequenced on an R9.4.1 flow cell (FLO-MIN106D) for 72 hours. The Dorado software (7.3.11), integrated into MinKNOW (v5.9.18), was used in real time for base calling sequencing reads using the High Accuracy model. The quality of the reads was evaluated using NanoPlot (v1.42.0) (3).

Overall, the sequencing generated 43,834 reads, with an N_50_ value of 12,958 bp (Table 1). Nanopore library adaptors were trimmed with Porechop software (v0.2.4) (4). The filtration by NanoFilt software (v2.8.0) (5) and removing of adaptors generated 35,699 reads. The long reads were assembled using the *de novo* assembler Flye (v2.9.3-b1797) (6), yielding two contigs. Polishing of the two contigs was performed using four rounds of racon (v1.5.0) (7) and a single round of medaka (v2.0.1) (8). The assembly statistics were generated using QUAST (v5.2.0) (9), while the completeness of the genomes was assessed using BUSCO (v5.5.0) (10). However, assembly using this strategy may contain a high number of errors and frameshifts. Default parameters were used for all software. The first contig constitutes a circular chromosome of 6,193,318 bp with 62.5% GC content. The second contig is a circular plasmid containing 394,003 bp with a GC content of 60.5%. The circularity was verified through assembly. The annotation was carried out using the NCBI Prokaryotic Genome Annotation Pipeline (PGAP) (v6.8) (11).

**TABLE 1:** Genome features of *Mesorhizobium* sp. strain AaZ16

Strain characteristic(s)	*Mesorhizobium* sp. AaZ16
Sampling site	Zaida lead and zinc mining site, Midelt, Morocco
Host plant	*Astragalus armatus*
No. of reads obtained	43,834
No. of reads (post-cleaning)	35,699
No. of contigs	2
Genome length (bp)	6,585,860
Coverage (×)	40
N_50_ (bp)	12,958
GC content (%)	62.51
BUSCO score (%)	95.1
Closest taxonomic assignation (TYGS)	*Mesorhizobium camelthorni*
dDDH formula 2 (GGDC) %	36
ANI value to closest species %	89.52
CDSs (coding DNA sequences)	6,485
No. of rRNAs	3
No. of tRNAs	52
No. of tmRNAs	1
BioSample ID	SAMN44267529
BioProject ID	PRJNA1172283
NCBI RefSeq assembly	GCF_043769475.1
GenBank ID	NZ_CP171729.1

The TYGS web-based platform (12) was used to determine the closest type strains, and the computational tool GGDC (13) was used to assess the genomic similarities between the two genomes, by determining DNA-DNA hybridization (DDH). The pyani command (v0.3.0-alpha) (14) allowed the validation of the results through the analysis of average nucleotide identity (ANI).

The sequencing results revealed that the strain belongs to the genus *Mesorhizobium* and shares 89.52% ANI value with the type strain *Mesorhizobium camelthorni* CCNWXJ404^T^. The dDDH revealed that strains AaZ16 and *M. camelthorni* CCNWXJ404^T^ share approximately 36% identity. The annotation of the AaZ16 genome by the NCBI pipeline PGAP revealed 6,485 coding DNA Sequences, 3 rRNA operons, 52 tRNAs, and 1 tmRNA (transfer-messenger RNA). As expected, the genes responsible for nodulation and nitrogen fixation are located in the chromosome as described in different rhizobial genera (15).

## Data Availability

The sequences corresponding to the genome of *Mesorhizobium* sp. AaZ16 have been deposited in GenBank under accession number NZ_CP171729.1, BioProject No. PRJNA1172283, and BioSample No. SAMN44267529. The raw sequencing reads are available in the Sequence Read Archive (SRA) under the accession number SRR30971815.
